# Real-time malaria detection in the Amazon rainforest via drone-collected eDNA and portable qPCR

**DOI:** 10.1016/j.onehlt.2025.101167

**Published:** 2025-08-20

**Authors:** Yin Cheong Aden Ip, Luca Montemartini, Jia Jin Marc Chang, Andrea Desiderato, Nicolás D. Franco-Sierra, Christian Geckeler, Mailyn Adriana Gonzalez Herrera, Michele Gregorini, Meret Jucker, Steffen Kirchgeorg, Martina Lüthi, Elvira Mächler, Frederik Bendix Thostrup, Guglielmo Murari, Marina Mura, Paola Pulido-Santacruz, Florencia Sangermano, Tobias Schindler, Claus Melvad, Stefano Mintchev, Kristy Deiner

**Affiliations:** a School of Marine and Environmental Affairs, University of Washington, Seattle, USA; b Diaxxo AG, Zürich, Switzerland; c Lee Kong Chian Natural History Museum, National University of Singapore, Singapore; d Department of Invertebrate Zoology and Hydrobiology, Faculty of Biology and Environmental Protection, University of Lodz, Łódź, Poland; e Corporación de Investigación e Innovación (VEDAS CII), VEDAS, Medellín, Antioquia, Colombia; f Department of Environmental Systems Science, Environmental Robotics Laboratory, ETH Zürich, Zürich, Switzerland; g Swiss Federal Institute for Forest, Snow and Landscape Research (WSL), Birmensdorf, Switzerland; h Alexander von Humboldt Biological Resources Research Institute, Bogotá 111311, Colombia; i Department of Environmental Systems Science, Ecosystems and Landscape Evolution, ETH Zürich, Zürich, Switzerland; j SimplexDNA AG, Winterthur 8404, Switzerland; k Institute for Mechanics and Production, Aarhus University, Aarhus, Denmark; l Indigenous Council of Inhaâ-bé, Amazon Region, Switzerland; m Escuela de Ciencias e Ingeniería, Universidad del Rosario, Bogotá 111221, Colombia; n Graduate School of Geography, Clark University, Worcester, MA 01566, USA; o Department of Environmental Systems Science, ETH Zürich, Zürich, Switzerland

**Keywords:** Indigenous communities, Plasmodium, Spillover, Surface swabbing, Zoonosis, Environmental DNA, Amazon rainforest, Mobile laboratory, One Health, Parasites

## Abstract

Zoonotic malaria risk at human-wildlife-environment interfaces requires surveillance that integrates signals from reservoirs, vectors and the environment. We coupled a drone-based environmental DNA (eDNA) canopy swabbing approach with portable quantitative PCR (qPCR) to detect *Plasmodium* DNA in situ during a 24-h field exercise in the Amazon rainforest. Drone-lowered sterile swabs into the canopy, which were then extracted and subjected to a multiplex pan-*Plasmodium* assay targeting five human-infecting *Plasmodium* species (limit of detection 0.2 parasites μL^−1^). Of 12 samples (10 canopy swabs, 2 field blanks; 13 total runs including repeats), one canopy swab amplified in duplicate (Ct = 28.7 and 29.23), while positive controls amplified as expected (Ct = 30.82 and 31.11) and all other environmental samples and blanks were negative. Passive acoustics confirmed co-occurring howler monkeys (*Alouatta* spp.), a known reservoir, whereas *Anopheles* mosquitoes were not recovered from concurrently deployed insect canopy traps. The end-to-end workflow, from drone deployment to qPCR diagnostic readout, averaged 1.5 h per assay, without requiring cold-chain logistics. This proof-of-concept demonstrates that intracellular parasite DNA can be recovered from canopy surfaces and read out in real-time, providing upstream, landscape-level intelligence to guide targeted vector surveillance in remote settings. Our approach operationalizes One Health by integrating environmental, wildlife, and vector signals within a single technological platform, representing a paradigm shift from reactive, sector-specific surveillance to proactive, integrated pathogen intelligence across the human-animal-environment interface.

## 1 Introduction

Emerging zoonotic parasites, particularly those transmitted between wildlife and humans, account for over 60 % of emerging infections [1], underscoring the need for enhanced surveillance strategies to mitigate future outbreaks [2,3]. The increasing frequency and magnitude of zoonotic spillovers, driven by anthropogenic activities such as deforestation, wildlife trade, and climate change, further amplify the urgency for integrated surveillance strategies [4]. Recent advances in molecular tools, such as real-time PCR, metagenomics, and environmental genomics, have enhanced our ability to detect and respond effectively to emerging novel infectious agents in wildlife, offering critical insights into the dynamics of infectious diseases [5]. Expanding surveillance capabilities is imperative, particularly in biodiverse, resource-limited, and even remote regions where the ecological interactions between parasites, pathogens, hosts, and vectors remain complex and poorly understood [6].

Traditional surveillance approaches often operate in institutional silos, with wildlife health, environmental monitoring, and public health agencies collecting data independently, limiting the ability to detect and respond to emerging spillover risks in real-time. The One Health approach addresses this by integrating wildlife reservoir monitoring, environmental pathogen detection, vector ecology assessment, and human health surveillance into coordinated early warning systems [7,8].

Malaria is a prime example of a vector-borne disease that continues to pose a significant global health threat [9]. Despite extensive eradication efforts, malaria remains prevalent, with an estimated 263 million cases and 597,000 deaths reported in 83 countries worldwide in 2023 [10]. The disease is caused by protozoan parasites of the genus *Plasmodium*, of which five species (*P. falciparum, P. vivax*, *P. ovale, P. knowlesi* and *P. malariae*) are responsible for causing disease in humans through transmission of bites of infected *Anopheles* mosquitoes. Timely identification of silent *Plasmodium* reservoirs is critical to inform vector-control operations and prevent recrudescence, particularly in hard-to-reach forested areas where routine entomological surveillance is logistically constrained [6]. In Latin America, most of the malaria burden is attributable to *P. vivax* [11]. Although the global burden of malaria has decreased in many regions, it continues to cause significant morbidity, mortality, and economic loss, particularly in tropical and subtropical areas such as South America [10,12]. The situation is especially critical in developing countries, where malaria prevention and control measures still face significant challenges [11,[13], [14], [15]].

The Amazon rainforest, a global biodiversity hotspot, presents a unique opportunity to study malaria dynamics due to its complex ecosystem and the reservoir potential of diverse host species. The Amazon basin has historically been a malaria-endemic region, with the state of Amazonas, Brazil, reporting one of the highest infection rates [16]. *Anopheles darlingi* is the primary vector, breeding in water bodies along river margins [17]. However, transmission dynamics are not uniform across the basin, with some areas being more prone to unstable or epidemic malaria and other regions experiencing a more stable or endemic transmission pattern (e.g. [18]). The Amazon's distinctive hydrology—comprising blackwater, whitewater, and clearwater rivers—plays a crucial role in shaping mosquito habitats and breeding sites [19]. Blackwater rivers, such as the Rio Negro, are known for their high acidity and low nutrient content, which can limit *Anopheles* mosquito breeding [20]. Likewise, deforestation exerts a significant influence on mosquito habitat suitability and malaria transmission potential in the Brazilian Amazon, but the relationship is complex. In some contexts, higher forest cover is associated with increased malaria risk due to greater vector habitat availability and human-vector contact at forest edges [21], while in others, deforestation facilitates mosquito proliferation by creating sunlit breeding sites and altering local ecology [22,23].

Given the need for effective surveillance in these ecologically diverse yet remote regions, our study employs drone-assisted environmental DNA (eDNA) sampling methods combined with portable molecular tools to enhance parasite detection and monitoring capabilities [[24], [25], [26], [27]]. Drones enable access to hard-to-reach areas, such as the forest canopy, where traditional sampling methods are often impractical or unsafe [28]. In addition, eDNA sampling provides a non-invasive approach to detect pathogens and parasites and monitor biodiversity by analyzing genetic material shed by organisms into the environment. Our eDNA sampling approach builds on established diagnostic capabilities from individual patient-level detection to landscape-level pathogen surveillance, enabling the simultaneous monitoring of parasite circulation across wildlife reservoirs, environmental compartments, and vector populations — the foundation of effective One Health surveillance [[29], [30], [31]]. Although *Plasmodium* spp. are intracellular, prior eDNA work shows protozoan fragments persist in water and on surfaces [32,33], providing the basis for our canopy-swab approach.

Building on this foundation, our study serves as proof of concept for the eDNA detection of *Plasmodium* spp. parasites—the causative agents of malaria—and their ecological relationships with potential reservoir hosts and mosquito vectors. Such an eDNA surveillance approach provides upstream, landscape-level pathogen intelligence relative to case-based detection [34,35]. Although eDNA cannot pinpoint individual source organisms [34], it provides critical upstream surveillance capabilities that detect pathogen circulation before spillover events occur, enabling preemptive public health responses in resource-limited settings [36]. Howler monkeys (*Alouatta seniculus*) have been implicated as key reservoirs in some regions [37], but other non-human primates, and potentially other wildlife, may also sustain parasite populations [17]. Understanding these broader host-vector-parasite interactions is essential for anticipating zoonotic spillover risks and informing public health surveillance strategies. Implementing One Health surveillance in remote, biodiverse ecosystems requires technological innovations that can simultaneously capture pathogen signals across multiple domains while enabling rapid, coordinated responses among wildlife health, environmental, and public health sectors. By combining remote eDNA sampling with drone deployment and portable parasite assays, we demonstrate a scalable, field-adaptable, framework for investigating malaria dynamics in remote and biodiverse environments. This integrative approach not only enhances early detection of emerging infectious diseases but also supports One Health strategies that account for the interdependence of environmental, animal, and human health [38].

## 2 Methods

### 2.1 Study area and tree canopy eDNA sample collection

**Fig. 1:**
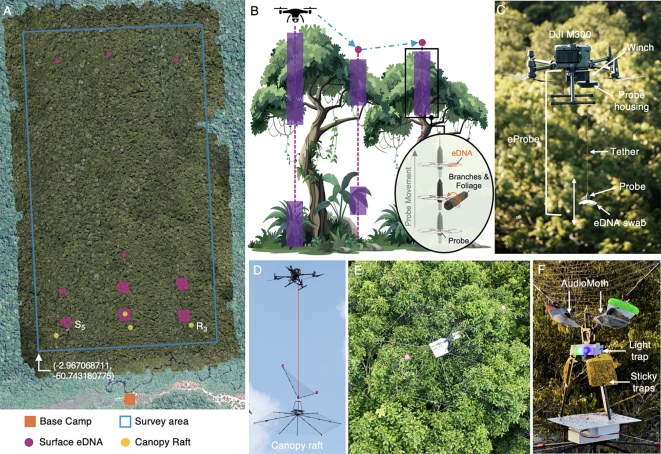
Overview of the survey technology and methodology. (A) Map of the 100-ha survey site (blue frame) and the base camp (orange square) used for drone deployments. Purple dots indicate locations where the eProbe was lowered into the canopy to collect eDNA, and yellow dots mark the deployment sites of the canopy rafts. (B, C) Operating principle of the eProbe: swabs are lowered into the canopy from a hovering drone using a robotic winch system. Surface eDNA is collected upon contact with vegetation. (D) Canopy raft transported by a drone for deployment atop the tree canopy (E). (F) The canopy raft integrates sensing and sampling instruments, including an AudioMoth acoustic recorder, light trap, and three sticky traps. This image was taken when the canopy raft was brought back for the overnight sampling highlighting the insects' specimens on the sticky traps. (For interpretation of the references to colour in this figure legend, the reader is referred to the web version of this article.)

The study was conducted at the XPRIZE competition test site within the Amazon rainforest in July 2024, a region characterized by its high biodiversity and complex ecological interactions. Sampling sites were located within a 1 km^2^ grid (0.75 × 1.34 km) centered at 2.9671° S, 60.7432° W (WGS 84) on the banks of the Rio Negro (Fig. 1A)—a blackwater river known for its high acidity and low nutrient levels, which can limit *Anopheles* mosquito breeding [20]. Access to genetic resources was registered in SISGEN under XPRIZE Rainforest - Equipe ETH BiodivX - Cadastro n° A0FB047. This study design aligns with One Health principles by integrating environmental pathogen surveillance (via eDNA), wildlife monitoring (acoustic detection), and vector surveillance (canopy traps) within a single technological platform to assess malaria transmission risk across ecological domains. In accordance with XPRIZE regulations, the biodiversity survey was conducted remotely using only robotic platforms and constrained to a 24 h deployment window, demonstrating the workflow's feasibility under strict time and logistical limits. Drones were employed for aerial imaging, surface eDNA collection from tree canopies (Fig. 1B and C), water eDNA sampling, and the deployment of canopy rafts. These rafts served as sensing platforms positioned above the canopy to collect overnight data via camera traps, audio recorders, and sticky traps (Fig. 1 D—F). While the present report focuses on surface eDNA collection and the data acquired with the canopy rafts, a comprehensive description of the full robot-based survey system is provided in [39].

A DJI Matrice 300 quadrotor equipped with custom-designed eDNA swabbing mechanisms (i.e., eProbe) was deployed to collect eDNA from the forest canopy layer (Fig. 1B), following the protocol described in [25]. The eProbe consists of a robotic winch mounted beneath the quadrotor, enabling the controlled lowering of a 60 g probe containing an eDNA swab, a circular fleece (Disko Lint-Free Fleece Cloths), into the canopy (Fig. 1C). During each sampling event, the quadrotor was flown over preselected GPS coordinates at the XPRIZE test site, while the probe was lowered into the vegetation using the tethered winch system. As the probe made physical contact with branches and foliage during both descent and ascent phases (see inset in figure Fig. 1B), it collected eDNA, including bodily fluids, feces, and other materials shed by organisms inhabiting or interacting with the canopy. Consequently, this approach allowed for the capture of eDNA from various vertebrates, such as arboreal primates and birds, as well as invertebrates like insects. A total of ten canopy swabs and two field blanks were collected during a systematic survey of a 100-ha area of rainforest (Fig. 1 A). Each sample collection required approximately 30 min of flight time. The number of samples was constrained by the 24-h survey window imposed by the competition. Sampling locations were primarily determined by drone safety and operational limitations. Sampling density was highest near the base camp (orange square in Fig. 1A), from which the drone was deployed, as these sites were within communication range of the remote controller and could be continuously monitored by the operator. Within this range, a single probe was lowered up to nine times every 30 m in a 100 × 100 m grid. The four most distant locations were sampled via fully autonomous operations and involved the highest operational risks. A detailed description of the sampling procedure during the XPRIZE competition is provided in [39].

To minimize contamination risks and ensure data integrity, strict protocols were followed in the field. Before and after each use, the eProbe housing was cleaned with 10 % bleach and wiped with deionized water, while a new pre-sterilized and individually packaged canopy swab was attached to the winch system. Prior to deployment, canopy swabs were dampened with 10 mL of individually packaged molecular grade DNA-free water before deployment to facilitate particle attachment to the material. Upon return from deployment, canopy swabs were placed into sterile 50 mL Falcon tubes immediately after retrieval, followed by eDNA extraction performed in situ, using a modified and rapid protocol optimized for eDNA extraction tailored for field conditions [25]. Finally, the resultant 5 mL volume of DNA extract was then concentrated twice using an Amicon® Ultra Centrifugal Filter, 30 kDa MWCO tube (following manufacturer's protocol) to a final 1 mL volume. The concentrated DNA was immediately subjected to further qPCR analysis.

### 2.2 Remote *Plasmodium* spp. qPCR detection

**Fig. 2:**
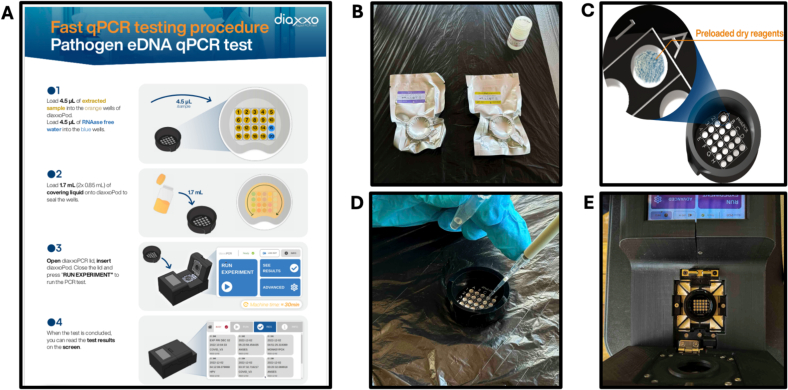
Portable qPCR setup using the Diaxxo system for remote, in situ, parasite detection from environmental DNA (eDNA) samples. (A) Schematic overview of the Diaxxo qPCR procedure, designed for fast and field-deployable detection of pathogens and parasites. The system can process up to 20 reactions simultaneously. (B) Vacuum-packed pods containing lyophilized mastermix and primers in each well, ensuring ease of transport to remote locations without the need for cold chain logistics. The sturdy packaging allows for easy transportation without risk of spills. (C) Close-up schematic of the lyophilized reagents in each well of the PCR pod, pre-loaded and ready for rehydration by adding the eDNA samples. (D) Addition of the eDNA extract into each well, where the sample liquid resuspends the lyophilized reagents and activates the mastermix. A layer of oil is added to prevent cross-contamination between wells, replacing the traditional heated lid used in laboratory thermocyclers. (E) The pod is loaded into the Diaxxo portable qPCR machine for amplification and analysis of the target parasite DNA.

The canopy eDNA extracts were screened for *Plasmodium* spp. using a portable Diaxxo AG qPCR system (Diaxxo AG, Switzerland) with lyophilized reagent pods (Fig. 2). The multiplex qPCR assay was designed by Diaxxo AG, which simultaneously targets five human-infecting *Plasmodium* species (*P. falciparum, P. vivax, P. malariae, P. ovale, and P. knowlesi*) [40]. Of these species, only *P. knowlesi* and *P. vivax* have primate reservoirs in nature. The assay's lower limit of detection, determined with PlasmoPod, was 0.2 parasites/μL [41,42]. Although originally validated for clinical samples, the assay's high sensitivity (0.2 parasites/μL) makes it suitable for environmental surveillance applications where parasite DNA concentrations are inherently lower. We applied a positivity cutoff of Ct < 35 to distinguish true parasite signals from degraded background eDNA, ensuring epidemiologically relevant detections. While formal spike-recovery validation was precluded by field deployment constraints, successful detection of positive controls (Ct 30.82–31.11) and environmental samples (Ct 28.7–29.23) demonstrates adequate extraction efficiency for surveillance applications. All reactions were run in duplicates to ensure reliability, and the entire amplification protocol took under 40 min, facilitating rapid field deployment.

Detection of *Plasmodium* spp. was achieved by targeting the conserved 18S rRNA gene, a region uniformly present across malaria parasites, using a pan-*Plasmodium* primer set adapted from previously validated assays [41,42]. The reaction mixture (proprietary) was prepared one week prior by Diaxxo AG, lyophilized on a qPCR pod (PlasmoPod; Fig. 2) and transported via 30 h flight from Switzerland to the test site in Brazil. After which, 5.5 μL of extracted DNA was added directly to each well of the qPCR pod (Fig. 2) following the manufacturer's protocol.

The thermocycling protocol was as follows: an initial incubation at 58 °C for 5 min, then an initial denaturation at 91 °C for 3 min, followed by an initial extension at 59 °C for 20 s. This was succeeded by 45 cycles of 90 °C denaturation for 12 s and 59 °C extension for 20 s. Images taken at the end of every qPCR cycle were analysed real-time and cycle threshold (Ct) values were recorded for each sample (Supplementary Material 1). Quality control was ensured through systematic testing across 13 qPCR runs using the same molecular-grade water batch for reagent rehydration, (10 eDNA canopy swab samples, 1 field blank, and 2 repeat runs of two eDNA samples). Positive controls confirmed assay functionality, with any Ct value below 35 considered positive for *Plasmodium* spp. This stringent threshold was chosen to ensure high specificity for environmental DNA samples, which have lower target concentrations and higher potential for non-specific amplification compared to clinical samples [41,42].

### 2.3 Insect identification from canopy rafts

In parallel with eDNA sampling, canopy rafts equipped with light traps, sticky traps, and acoustic recorders (AudioMoth) were deployed by a DJI Matrice 300 on the upper forest canopies to survey insect biodiversity (Fig. 1 D-F; [39]). Each raft was attached to the drone via a 3 m tether designed to self-disengage once the raft's weight was fully supported by the tree canopy. Deployment locations consisting of dense, flat tree canopies were identified by scouting the competition area with an imaging drone (DJI Mavic 4). Retrieval involved attaching a treble fishing hook to the tether and teleoperating the drone to engage the net on top of the raft. While this deployment and retrieval procedure proved robust, it required precise drone teleoperation. Consequently, the four rafts were positioned in close proximity to the base camp, where drone connectivity was optimal (Fig. 1A). All four canopy rafts with traps were deployed in the afternoon and left in place for 12 h before retrieval at dawn the following morning.

Upon retrieval, insect specimens were sorted and identified under a microscope, with particular attention to mosquito species, especially those belonging to the *Anopheles* genus, which are known vectors for malaria transmission. Each specimen was carefully removed from the traps using sterile tweezers and placed in Petri dishes for further processing, including imaging and DNA barcoding. Insect specimens were ultimately preserved in ethanol. It should be noted that light traps and sticky traps are not optimized for *Anopheles* mosquito capture, which typically requires CO₂-baited traps or human landing catches. Our insect surveillance was originally intended for broad-based general biodiversity assessment rather than targeted vector surveillance.

DNA extraction was performed via the non-destructive HotSHOT (hot sodium hydroxide and Tris) method [43]. The HotSHOT procedure yields PCR-ready DNA when using very small quantities of tissue [44]. Small insects (< 1 cm) were immersed directly into the lysis buffer and tissue from big insects (> 1 cm long) was obtained by separating the tibia and tarsomere from the hind legs of each individual using sterile blades. DNA extraction was carried out using 40 μL of lysis buffer (25 mM NaOH, 0.2 mM EDTA, pH 8) and then incubated at 65 °C for 18 min followed by 98 °C for 2 min. Thereafter, 40 μL of neutralization solution (40 mM Tris-HCl) was added to each sample.

The LCO1490 and HCO2198 [45] primer pair was used to amplify a 658 bp fragment of the COI gene. Each primer contained an additional 13 bp tag on the 5′-end for multiplexing numerous samples for sequencing [46]. PCRs were performed using Cytiva PuRe Taq Ready-To-Go PCR beads in 0.2 ml tubes (8 tube strips) with 2 μL of template DNA, 0.5 μl each of 10 μM tagged primers and 22 μL of molecular grade water to complete a final volume of 25 μL. PCR products were pooled (1 μL per reaction) and purified using AMPureXP beads at 0.75× ratio. The cleaned-up pool was then used for the sequencing library preparation following [46]. The library was sequenced on an Oxford Nanopore MinION MK1b device. Consensus DNA barcodes were obtained with ONTbarcoder v2.1.3 [47] and subsequently compared to the BOLD database for taxonomic assignment.

### 2.4 Acoustic detection of primates

Passive acoustic monitoring was conducted using AudioMoth recorders [48] integrated into the canopy rafts (Fig. 1F) to capture vocalizations of animal species living in the forest for biodiversity assessment (Supplementary Fig. 1). The recorders were programmed to capture sound continuously using a sampling rate of 46 kHz and were processed using BirdNET Plus Analyzer [49] with a custom classifier developed by Rainforest Connection that included howler monkeys in its training. Although seven species of primates are found in the area [50], howler monkeys (*Alouatta seniculus*) were the only primates included in the database. Given this limitation of the supervised audio species identification, we also performed a manual extraction of calls potentially belonging to primates, which were then validated by a local indigenous scientist from Inhaa-be. Since howler monkeys are known to be potential reservoirs for *Plasmodium* spp., the acoustic data were then cross-referenced with the qPCR results to highlight any correspondence between the presence of howler monkeys and *Plasmodium* spp. DNA in the same area.

## 3 Results

### 3.1 Rapid field detection of *Plasmodium* spp.

**Fig. 3:**
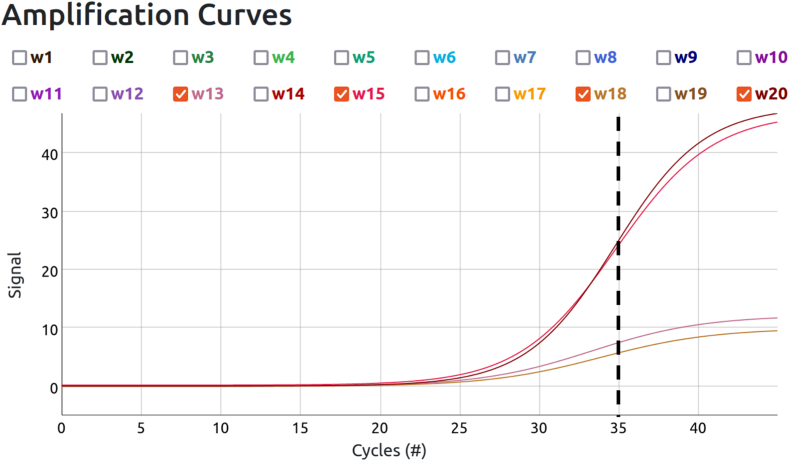
Amplification curves of the surface swab eDNA sample S_005 for the *Plasmodium* spp. assay, showing detection in wells 13 and 18 with Ct values of 28.7 and 29.23, respectively (lower curves). The positive process control duplicate assays, loaded with molecular-grade water, are shown in wells 15 and 20 with Ct values of 30.82 and 31.11, respectively (upper curves). The black vertical dashed line indicates the detection threshold (Ct < 35). These results confirm the detection of *Plasmodium* spp. in the canopy eDNA sample while validating the reliability of the qPCR assay.

The integrated One Health surveillance workflow, combining environmental pathogen detection, wildlife monitoring, and vector assessment, delivered actionable intelligence within 1.5 h per assay (approximately 30 min for drone flight, 20 min for DNA extraction, and 40 min for qPCR), enabling rapid cross-sector decision-making. Subsequently, qPCR analysis of eDNA samples from the canopy confirmed the presence of *Plasmodium* spp. in one canopy swab sample. Amplification curves from wells 13 and 18, which were PCR duplicates, produced consistent Ct values of 28.7 and 29.23 (Fig. 2; Supplementary Material 1), confirming *Plasmodium* spp. detection in the sample collected from an area with documented howler monkey activity. Concurrently, the positive control in wells 15 and 20 amplified as expected, with Ct values of 30.82 and 31.11, while the negative field blank control run showed no amplification (Fig. 3). The conservative Ct threshold of <35 ensured high confidence, minimizing the risk of false positives from environmental contaminants.

Quality control across the complete dataset confirmed assay specificity and absence of contamination. Of 13 total runs using the same reagent batch, only sample S_005 showed *Plasmodium* detection, while 11 environmental samples and one field blank water-only control remained negative for *Plasmodium* (Supplementary Material 1).

### 3.2 Acoustic confirmation of howler monkey presence

Vocalizations belonging to howler monkeys (*A. seniculus*) were identified in the recordings from raft number 3 (Fig. 1A). Automated identification of *A. seniculus* resulted in false positive detections where other low-frequency sounds, mainly from motorboats, were identified as vocalizations of this species. The quality of segments automatically detected by the classifier was not sufficient for confirmation of the presence of the reservoir. However, manually extracted audio segments were confirmed to belong to both adult and infant *A. seniculus* individuals (Supplementary Audio 1, B), allowing the confirmation of the presence of this species in the study area.

### 3.3 Mosquito surveillance

Visual identification and DNA barcoding of the captured insects revealed a diverse range of species, with *Lepidoptera* (moths) being the most abundant (Supplementary Fig. 2). A total of 45 insect species were identified. No *Anopheles* mosquitoes were detected in the canopy traps.

## 4 Discussion

### 4.1 Canopy eDNA surveillance as an early-warning tool

By confirming that intracellular-parasite DNA can be recovered from canopy swabs—building on established eDNA detections of protozoa in water and on surfaces [32,33]—we demonstrate a viable early-warning tool for malaria surveillance. The end-to-end workflow (≈30 min flight, 20 min extraction, 40 min qPCR) yielded results in ∼1.5 h, enabling rapid field decisions. Integrated into national programs via Ct-threshold triggers (e.g., Ct < 30 vector-control mobilization), this field-deployable drone-qPCR delivers actionable data in under two hours. While this approach enables rapid pathogen detection, its application in large endemic regions like the Amazon requires strategic deployment at transmission hotspots rather than region-wide coverage, serving as an early warning system to guide targeted vector control interventions. This follows established precedents in environmental pathogen surveillance, including wastewater-based COVID-19 monitoring and West Nile virus vector pool testing, which provide landscape-level pathogen intelligence for public health decision-making [[51], [52], [53], [54]].

Our technological approach operationalizes core One Health principles by breaking down traditional surveillance silos. Rather than separate teams working independently, our platform provides integrated pathogen intelligence accessible to all sectors simultaneously. Moreover, the 1.5-h workflow enables coordinated responses where wildlife health agencies can assess reservoir populations, environmental health teams can implement targeted monitoring, and public health authorities can activate community preparedness measures based on shared pathogen detection data.

While eDNA detection cannot identify specific source organisms [34], the epidemiological advantages lie in its capacity for landscape-level risk assessment and to act as an early warning system. Our approach has inherent limitations common to eDNA surveillance: source organisms cannot be definitively identified, and parasite viability cannot be confirmed. However, these limitations must be weighed against the epidemiological value of landscape-level pathogen surveillance in resource-limited settings.

Despite these limitations, the integration of eDNA sampling with drone technology and portable qPCR assays provides a powerful framework for monitoring species interactions and parasite dynamics in previously inaccessible areas of the rainforest. The apparent absence of mosquitoes in canopy traps, coupled with the presence of *Plasmodium* in eDNA swabs, underscores the importance of vector distribution in shaping disease dynamics. The rapid nature of our workflow with drone-based eDNA sampling, combined with portable qPCR assays, highlights the transformative potential of portable, real-time and non-invasive eDNA technology for landscape-level parasite surveillance, especially in remote and resource-limited settings.

Seasonal variations in primate movement, parasite prevalence, and eDNA persistence could influence detection outcomes. This proof-of-concept demonstrates technical feasibility; operational implementation requires longitudinal, multi-seasonal sampling campaigns to establish baseline pathogen circulation patterns and validate surveillance signals across environmental conditions.

### 4.2 Ecological and epidemiological insights

**Fig. 4:**
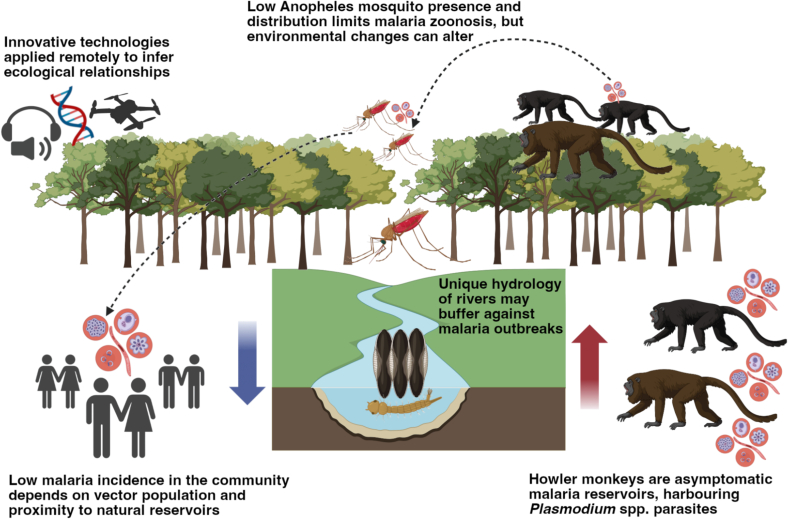
This illustration highlights the ecological relationships inferred using innovative technologies, including drone-based eDNA sampling, portable qPCR assays for *Plasmodium* detection, DNA barcoding of insect traps, and audiometry to confirm howler monkey presence. Our results show no detection of *Anopheles* mosquitoes, consistent with known data that howler monkeys (*Alouatta seniculus*) act as natural reservoirs for *Plasmodium* spp. in the Amazon. We propose that the current low malaria risk in the region is due to the scarcity of *Anopheles* mosquitoes, underscoring the importance of enhanced biodiversity monitoring for proactive disease prevention. (Icons were generated using biorender.com)

Understanding the intricate ecological relationships in the Amazon rainforest is essential for unraveling the dynamics of zoonoses like malaria (Fig. 4). More importantly, real-time detection in the canopy can feed directly into public-health decision-making, providing early warning of parasite presence upstream of human cases.

The detection of *Plasmodium* eDNA in canopy swabs suggests parasite presence in arboreal wildlife, likely through bodily fluids or fecal matter. This finding aligns with prior studies identifying howler monkeys (*Alouatta seniculus*) as asymptomatic carriers of *Plasmodium* spp. in Latin America [37]. While other wildlife species in the Amazon ecosystem may also contribute to environmental *Plasmodium* signals, the spatial correlation with howler monkey presence provides biological validation of active pathogen circulation in wildlife reservoirs. However, canopy entomological surveillance more commonly employs CO₂-baited traps or human landing catches, which are generally more effective for host-seeking *Anopheles* spp. than the light traps used in our study [6]. Our 24-h XPRIZE competition timeframe and focus on general biodiversity assessment also limited the temporal scope of our findings. Consequently, the apparent absence of *Anopheles* in our light-based canopy traps suggests low vector densities in the canopy rather than confirming their complete absence. The spatial overlap between confirmed howler monkey calls and the positive canopy swab supports reservoir-associated circulation at the sampled site. Coupled with the apparently absent vector populations, this provides critical intelligence for public health decision-making. Together they indicate: (1) active parasite circulation in wildlife reservoirs, (2) low immediate transmission risk due to vector scarcity, and (3) potential spillover vulnerability should environmental conditions change.

This integrated surveillance approach exemplifies One Health in practice by providing shared pathogen intelligence that informs coordinated decision-making across sectors. Wildlife health agencies can prioritize primate population monitoring, environmental health teams can intensify water quality surveillance, vector control programs can optimize resource deployment, and public health authorities can implement targeted community preparedness—all triggered by the same eDNA detection event.

While we cannot determine infection status of individual organisms, we can assess landscape-level pathogen circulation—information that is equally valuable for public health planning and often more actionable than waiting for human cases to emerge. The ecological context of our study further supports this surveillance approach. The hydrological characteristics of blackwater rivers in the sampling area, like the Rio Negro, create suboptimal breeding conditions for *Anopheles* species, including *Anopheles darlingi*, the primary malaria vector in the region [19,20]. These environmental factors serve as natural barriers preventing zoonotic spillover, suggesting that vector suppression, rather than parasite absence, can drive malaria transmission dynamics in this ecosystem, with spillover occurring only under specific environmental conditions. Additionally, shifting transmission risk in response to climate change, with rising temperatures and altered rainfall patterns, may allow the malaria-carrying mosquitoes to thrive in once unsuitable areas. Ongoing environmental changes, such as deforestation and global warming, could further exacerbate this, potentially increasing mosquito populations and leading to future malaria outbreaks [55,56].

### 4.3 One health implementation and future directions

The accelerating pace of environmental change in the Amazon—with Brazil losing 3.07 million hectares of natural forest in 2023 alone [46]—exemplifies why One Health approaches are essential for zoonotic disease prevention. Traditional sector-specific responses cannot adequately address the complex, interconnected drivers of disease emergence that span wildlife habitat fragmentation, vector ecology shifts, and human exposure patterns. Our drone-eDNA surveillance platform demonstrates how technological innovation can operationalize One Health principles by providing integrated pathogen intelligence that supports coordinated multi-sector responses.

Implementation of this One Health surveillance approach requires institutional frameworks that facilitate data sharing and coordinated responses across wildlife health, environmental monitoring, and public health agencies. We propose establishing regional One Health surveillance networks where eDNA detection triggers activate standardized response protocols: wildlife health teams initiate targeted reservoir species monitoring, environmental health agencies intensify ecosystem surveillance, vector control programs deploy enhanced trapping, and public health authorities activate community preparedness measures. Such coordinated responses maximize the epidemiological value of environmental surveillance while ensuring efficient resource utilization across sectors.

Future research should establish One Health surveillance networks integrating seasonal eDNA monitoring with coordinated wildlife health, vector ecology, and human health surveillance programs. Long-term implementation requires developing institutional frameworks for cross-sector data sharing, standardized response protocols, and collaborative funding mechanisms that recognize the shared benefits of integrated surveillance. While demonstrated here for malaria in Amazonian ecosystems, this One Health surveillance approach could be adapted for other vector-borne pathogens and ecological contexts, providing a scalable framework for addressing the interconnected health challenges of the Anthropocene.

In conclusion, our findings suggest that howler monkeys (*Alouatta seniculus*) and potentially other non-human primates may serve as asymptomatic reservoirs for *Plasmodium* spp. in parts of the Amazon. However, the apparent limited presence of *Anopheles* mosquitoes remains the primary ecological barrier preventing malaria transmission to humans in these regions. Maintaining forest integrity and minimizing human disturbances are, therefore, crucial for mitigating zoonotic malaria risks. By establishing a rapid, drone-assisted parasite detection workflow with in-situ qPCR readout, we demonstrate a field-deployable platform capable of triggering proactive vector-control and outbreak-response measures—fulfilling a critical need in remote, high-risk regions. This work provides a practical model for implementing One Health approaches through coordinated protocols between wildlife health, vector control, and public health agencies, with eDNA detection thresholds triggering multi-sector responses. While developed for malaria surveillance in arboreal ecosystems, the underlying approach could potentially be adapted for other vector-borne pathogens, providing a scalable framework for addressing interconnected health challenges.

## Credit authorship contribution statement

**Yin Cheong Aden Ip:** Writing – review & editing, Writing – original draft, Visualization, Validation, Methodology, Investigation, Formal analysis, Data curation, Conceptualization. **Luca Montemartini:** Writing – review & editing, Validation, Methodology, Formal analysis, Data curation, Conceptualization. **Jia Jin Marc Chang:** Writing – review & editing, Visualization, Validation, Data curation, Conceptualization. **Andrea Desiderato:** Writing – review & editing, Methodology, Investigation, Data curation. **Nicolás D. Franco-Sierra:** Writing – review & editing, Visualization, Validation, Methodology, Data curation. **Christian Geckeler:** Writing – review & editing, Resources, Methodology, Conceptualization. **Mailyn Adriana Gonzalez Herrera:** Visualization, Validation, Resources, Methodology, Data curation. **Michele Gregorini:** Validation, Software, Resources, Methodology, Conceptualization. **Meret Jucker:** Writing – review & editing, Resources, Methodology, Data curation. **Steffen Kirchgeorg:** Writing – review & editing, Software, Resources, Methodology, Conceptualization. **Martina Lüthi:** Writing – review & editing, Validation, Methodology, Investigation. **Elvira Mächler:** Resources, Project administration, Methodology, Investigation. **Frederik Bendix Thostrup:** Resources, Methodology. **Guglielmo Murari:** Software, Resources, Methodology. **Marina Mura:** Validation, Data curation. **Paola Pulido-Santacruz:** Writing – review & editing, Validation, Resources, Methodology, Investigation. **Florencia Sangermano:** Writing – review & editing, Visualization, Validation, Software, Resources, Methodology, Formal analysis. **Tobias Schindler:** Validation, Software, Resources, Methodology. **Claus Melvad:** Supervision, Resources, Methodology. **Stefano Mintchev:** Writing – review & editing, Visualization, Validation, Supervision, Software, Resources, Project administration, Methodology, Investigation, Funding acquisition, Data curation. **Kristy Deiner:** Writing – review & editing, Visualization, Validation, Supervision, Project administration, Methodology, Investigation, Funding acquisition, Data curation.

## Declaration of competing interest

This study was funded by the Rütli-Stiftung, the ETH Foundation, the XPRIZE Foundation, and the Alana Foundation via the participation of the authors in the XPRIZE Rainforest competition. S.M., S.K., and C.G. were supported by the 10.13039/501100001711Swiss National Science Foundation through the Eccellenza Grant (grant number 186865). All data are available in the main text and supplementary data.

M.G., L.M., G.M. and T.S. are employed by, and shareholders of, the ETH Zurich spin-off company—Diaxxo AG—the organization that produces the DiaxxoPCR device. The Method and UAV for collecting environmental DNA is protected for commercial use under the patent WO2024246233A1. K.D. and E.M. are the owners of SimplexDNA, a company that provides eDNA services in a commercial setting. All other authors declare that they have no competing interests. Access to genetic resources was registered in SISGEN under XPRIZE Rainforest - Equipe ETH BiodivX - Cadastro n° A0FB047. No institutional ethics approval was required as the study involved only environmental DNA collection and acoustic monitoring without direct animal contact, and sample collection from invertebrate insects.

## Data Availability

Data will be made available on request.

## Appendix A Supplementary data

Supplementary material - Audio 1A and B. *Alouatta seniculus* adult and infant field audiomoth recording. Image 1

Supplementary material Audio 2 Image 2

Supplementary material 1 qPCR raw data. Image 3

Supplementary Figure 1. External audiomoths attached to the deployment raft. Supplementary Figure 2. DNA barcoding of captured insects revealed a diverse range of species, with Lepidoptera (moths) being the most abundant (n=83). A total of 45 species were identified, though no malaria vectors were found, consistent with the absence of mosquitoes in the sticky traps. Image 4
