# Supplementary material for: Real-time malaria detection in the Amazon rainforest via drone-collected eDNA and portable qPCR
Source: One Health. 2025 Aug 20;21:101167. doi: 10.1016/j.onehlt.2025.101167 (PMC12398916; doi:10.1016/j.onehlt.2025.101167)
Supplement: Supplementary file 4 — Supplementary Figure 1. External audiomoths attached to the deployment raft. Supplementary Figure 2. DNA barcoding of captured insects revealed a diverse range of species, with Lepidoptera (moths) being the most abundant (n=83). A total of 45 species were identified, though no malaria vectors were found, consistent with the absence of mosquitoes in the sticky traps. [file mmc4.pdf]

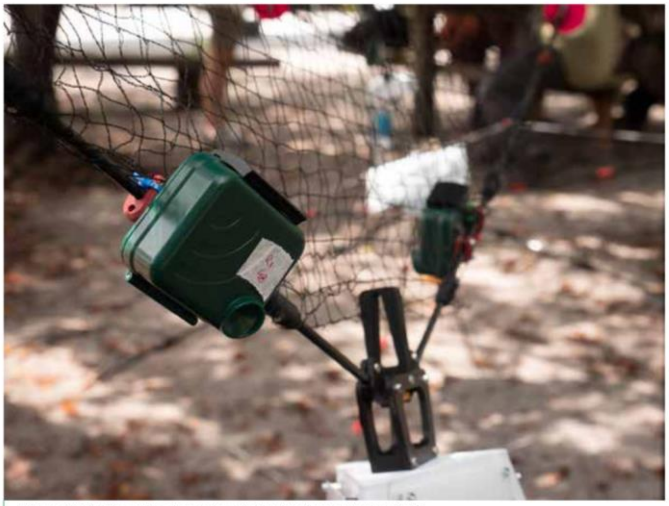

Figure 4.2.16: External audiomoths attached to the deployment raft.

Supplementary Figure 1. External audiomoths attached to the deployment raft.

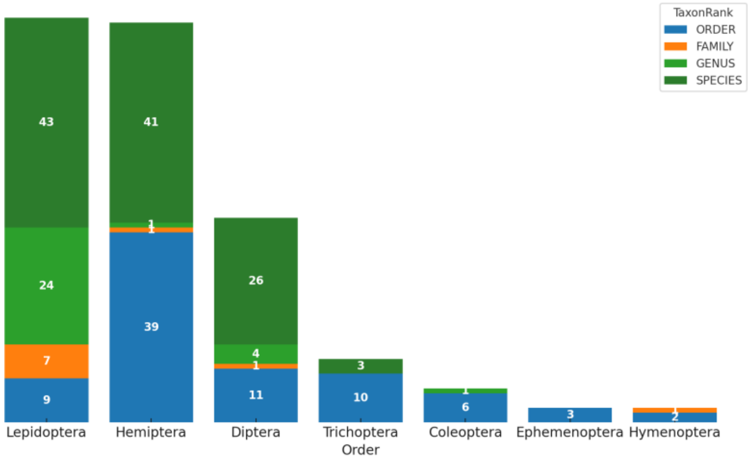

Supplementary Figure 2. DNA barcoding of captured insects revealed a diverse range of species, with Lepidoptera (moths) being the most abundant (n=83). A total of 45 species were identified, though no malaria vectors were found, consistent with the absence of mosquitoes in the sticky traps.
